# Effect of ABO blood group on the severity and clinico-pathological parameters of COVID-19

**DOI:** 10.12669/pjms.40.5.9037

**Published:** 2024

**Authors:** Lareb Asad, Talat Mirza, Santosh Kumar, Ambrina Khatoon

**Affiliations:** 1 Dr. Lareb Asad. Ziauddin University Hospital, Karachi, Pakistan; 2 Dr. Talat Mirza, Meritorious Professor, Department of Pathology, Executive Director (Research), Ziauddin University Hospital, Karachi, Pakistan; 3 Dr. Santosh Kumar, Associate Professor, Department of Pathology, Ziauddin University Hospital, Karachi, Pakistan; 4 Dr. Ambrina Khatoon, Assistant Professor, Department of Molecular Medicine, Ziauddin University Hospital, Karachi, Pakistan

**Keywords:** ABO Blood Group, COVID-19, Severity, Clinico-pathological Parameters

## Abstract

**Background and Objective:**

The COVID-19 pandemic has highlighted the need to understand the factors affecting disease severity. Prior research has indicated the potential roles of the ABO blood group system in disease susceptibility and progression. Our objective was to investigate the association between ABO Blood groups and the severity of COVID-19 and clinicopathological parameters.

**Methods:**

An analytical cross-sectional study was conducted across three locations of Ziauddin University Hospital, including COVID-19 outpatient departments (OPDs), wards, and intensive care units (ICUs) from May 2020 to December 2020.The study utilized a non-probability convenient sampling technique with a sample size of 120 PCR-positive adult patients, as calculated by OpenEpi with a 95% confidence interval. Patients were excluded if they were under 14, intellectually impaired, post-chemotherapy or radiotherapy, or had a malignant condition. Disease severity was determined based on clinicopathological parameters and associated with blood group data using ANOVA and Chi-square tests in SPSS version 21.

**Results:**

A significant association was found between the ABO blood groups and COVID-19 severity. Blood group-A was notably overrepresented in patients with severe COVID-19 and correlated with higher inflammatory markers and coagulation parameters.

**Conclusion:**

ABO blood group, particularly Blood Group-A significantly associates with the severity of COVID-19. This finding suggests the potential utility of ABO blood group typing as a predictive marker for disease severity, which could contribute to personalized patient management strategies. Further research is necessary to understand the mechanisms underlying this association.

## INTRODUCTION

Since December 2019, an outbreak of pneumonia caused by a novel coronavirus emerged in Wuhan, Hubei Province. The disease was later named coronavirus disease 2019 (COVID-19) by the World Health Organization (WHO). The virus has spread to various parts of China and other countries. It is related to the viruses responsible for severe acute respiratory syndrome (SARS) and Middle East respiratory syndrome (MERS). The world is suffering a heavy toll from the COVID-19 pandemic, with over 191 million confirmed cases and nearly 4.1 million deaths globally; irrespective of age and sex, especially in older adults with comorbidities, such as cardiovascular, cerebrovascular diseases and diabetes. In Pakistan, By 16 August 2023, there have been 1,580,631 confirmed cases of COVID-19 with 30,656 deaths, reported to WHO. As of 19 August 2023, a total of 339,946,677 vaccine doses have been administered (https://covid19.who.int).

**Table-I:** Association of clinicopathologic parameters of Covid-19 with bloods groups.

Parameters	Total No. Of Patients	Blood Group					P value

		A+ve	AB+ve	B+ve	O-ve	O+ve	
***Age***							
>50	89	48(40%)	10(8.3%)	26(21.7%)	3(2.5%)	2(1.7%)	0.088
≤50	31	14(11.7%)	6(5%)	7(5.8%)	0(0%)	4(3.3%)	
***Gender***							
Male	65	33(27.5%)	8(6.7%)	17(14.2%)	3(2.5%)	3(2.5%)	0.597
Female	56	29(24.2%)	8(6.7%)	16(13.3%)	0(0%)	3(2.5%)	
***Ethnicity***							
Sindhi	31	18(15%)	2(1.7%)	6(5%)	1(0.8%)	3(2.5%)	0.815
Urdu	67	34(28.3%)	10(8.3%)	20(16.7%)	1(0.8%)	2(1.7%)	
Punjabi	15	6(5%)	3(2.5%)	4(3.3%)	1(0.8%)	1(0.8%)	
Pathan	8	4(3.3%)	1(0.8%)	3(2.5%)	0(0%)	0(0%)	
Severity							
***Asymptomatic***	16	4(3.3%)	2(1.7%)	7(5.8%)	0(0%)	2(1.7%)	0.264
Mild	11	5(4.2%)	2(1.7%)	2(1.7%)	1(0.8%)	1(0.8%)	
Moderate	32	15(12.5%)	2(1.7%)	13(10.8%)	1(0.8%)	1(0.8%)	
Severe	38	24(20%)	5(4.2%)	6(5%)	1(0.8%)	2(1.7%)	
Critical	24	14(11.7%)	5(4.2%)	5(4.2%)	0(0%)	0(0%)	
***DM***							
Yes	61	32(26.7%)	4(3.3%)	20(16.7%)	2(1.7%)	3(2.5%)	0.211
No	59	30(25%)	12(10%)	13(10.8%)	1(0.8%)	3(2.5%)	
***HTN***							
Yes	71	38(31.7%)	4(3.3%)	23(19.2%)	3(2.5%)	3(2.5%)	0.02
No	49	24(20%)	12(10%)	10(8.3%)	0(0%)	3(2.5%)	
***Fever***							
Yes	77	41(34.2%)	10(8.3%)	20(16.7%)	2(1.7%)	3(2.5%)	0.939
No	43	21(17.5%)	5(4.2%)	13(10.8%)	1(0.8%)	3(2.5%)	
***Cough***							
Yes	50	27(22.5%)	6(5%)	14(11.7%)	1(0.8%)	1(0.8%)	0.769
No	71	35(29.2%)	10(8.3%)	19(15.8%)	2(1.7%)	5(4.2%)	
***SOB***							
Yes	71	41(34.2%)	8(6.7%)	19(15.8%)	1(0.8%)	2(1.7%)
No	49	21(17.5%)	8(6.7%)	14(11.7%)	2(1.7%)	4(3.3%)
***Body Ache***							
Yes	18	7(5.8%)	3(2.5%)	5(4.2%)	0(0%)	2(1.7%)	0.552
No	103	55(45.8%)	13(10.8%)	28(23.3%)	3(2.5%)	4(3.3%)	
***Loss of Taste***							
Yes	4	1(0.8%)	0(0%)	1(0.8%)	1(0.8%)	1(0.8%)	0.012
No	116	61(50.8%)	16(13.3%)	32(26.7%)	2(1.7%)	5(4.2%)	
***Loss of Smell***							
Yes	6	2(1.7%)	1(0.8%)	1(0.8%)	1(0.8%)	1(0.8%)	0.111
No	114	60(50%)	15(12.5%)	32(26.7%)	2(1.7%)	5(4.2%)	
***Gen. weakness Weakness***							
Yes	29	13(10.8%)	5(4.2%)	8(6.7%)	1(0.8%)	1(0.8%)	0.896
No	92	49(40.8%)	11(9.2%)	25(20.8%)	2(1.7%)	5(4.2%)	
***Outcome***							
Discharged	98 =SUM(RIGHT)	45(37.5%)	14(11.7%)	30(25%)	3(2.5%)	6(5%)	0.104
Death	22	17(14.2%)	2(1.7%)	3(2.5%)	0(0%)	0(0%)	

Early reports indicated that most COVID-19 cases were linked to the Hunan Seafood Market. Common clinical symptoms included fever, cough, difficulty breathing, myalgia, fatigue, altered white blood cell count, and evidence of pneumonia on imaging.^1^

SARS-CoV-2, a member of the Coronaviridae family, primarily spreads through respiratory droplets, causing symptoms ranging from asymptomatic infection to severe pneumonia and death.^2^,^3^ Known risk factors include age, male gender, and coexisting conditions such as hypertension, diabetes, and cardiovascular disease.^4^ However, host genetic factors are increasingly being recognized for their role in dictating disease susceptibility and severity, including the ABO blood group system.^5^

The ABO system, classified into four main types (A, B, AB, and O) based on specific antigens on red blood cells, is also expressed on many other cells, including those in the respiratory and gastrointestinal tract where SARS-CoV-2 enters. Previous studies indicate an association between certain blood groups and various disease susceptibilities. (for example Hep B virus, Norwalk virus, or bacterial diseases like *H.pylori*.^6^

This association is believed to result from the effect of ABO antigens on host-pathogen interactions. Early observational studies indicate a potential link between ABO blood group and COVID-19 susceptibility and severity, with blood Group-A associated with increased risk and O with lower risk.^7^,^8^ However, these findings are inconsistent across all studies and the underlying mechanisms remain unclear.^5^,^9^,^10^

At the peak of the pandemic, there were mass fatalities and scientists have been trying to unfold every aspect of the deadly virus that killed thousands of people making it an urgent medical issue for a solution. Our Objective was to investigate the association between ABO Blood groups and the severity of COVID-19 and clinicopathological parameters.

## METHODS

This cross-sectional study was conducted across three locations of Ziauddin University Hospital from May 2020 to December 2020, including COVID-19 outpatient departments (OPDs), wards, and intensive care units (ICUs). The study utilized non-probability convenient sampling technique with a sample size of 120 PCR-positive adult patients.

### Exclusion Criteria

Patients were excluded if they were under 14, intellectually impaired, post-chemotherapy or radiotherapy, or had a malignant condition.

### Inclusion Criteria

PCR-positive patients aged fourteen or above of either gender from inpatients department or Intensive Care Unit at Ziauddin Hospital Clifton, KDLB, and Nazimabad destinations Karachi with varying severity of the disease were included in our study. Using aseptic techniques 5ml of blood was drawn and transferred to tubes containing ethylene diamine tetraacetic acid (EDTA) blood tests conducted concurrently. Using the slide method, the antigen-antibody agglutination test was carried out to ensure blood grouping and Rhesus factor. The Rhesus (Rh) factor was determined using the ABO monoclonal reagents, which are hybridized immunoglobulins IgM + IgG.

### Statistical Evaluation

Disease severity categorized by WHO was determined based on Clinicopathological parameters and correlated with blood group data using ANOVA and Chi-square tests in SPSS version 21 Clinicopathological characteristics such as PCR findings, CBC counts, CRP, and other numeric variables with the severity of COVID were assessed by applying ANOVA. A Chi-Square test was performed to check the association between ABO Blood Groups with disease severity. A P-value less than 0.05 was considered significant (https://apps.who.int/iris/bitstream/handle/10665/349321/WHO-2019-nCoV-clinical-2021.2-eng.pdf).

### Ethical Approval

Ethical approval was given by the “Ethics Review Committee (ERC)” of Ziauddin University before the commencement of the study. (Reference code; 5950922LAPAT).

### Participant consent

all patients were informed that their participation was voluntary, and consent was obtained before the study.

## RESULTS

Out of 120 patients, 62 patients were of Blood Group-A+ve, 33 B+ve, 16 AB+ve, three O-ve, and six O+ve. There were no significant differences in age or gender between the blood types. Hypertension and loss of taste had the significant association with the blood groups with p-value of 0.02 and 0.012 respectively. These patients also exhibited elevated inflammatory markers and coagulation parameters. Only LDH levels were found to be significant with the P value of 0.005.

Furthermore, current study also analyzed the association of clinicopathological parameters with the severity of disease. Out of a total number of 120 patients, 24 (20%) patients were critically ill, 38 (26.7 %) were severe cases. Cough, fever, loss of taste, body ache, and shortness of breath found to be associated with severity, whereas in laboratory parameters D- dimer, LDH, ferritin, and CRP levels were also found significant. Blood group A+ve was found to be more prevalent in severe 38 (31.7%) cases followed by blood group B+ve 11(9.2%), and AB+ve 10(8.4%).

## DISCUSSION

Our study found a significant association between Blood Group-A and the severity of COVID-19. The link between ABO blood groups and disease susceptibility, especially in infectious diseases, has been the focus of multiple studies in the past and continues to be explored. With the onset of the COVID-19 pandemic, it has been more pressing to understand the determinants of disease susceptibility and severity.^11^ Epidemiological studies suggest that people with certain blood group types may have a higher susceptibility to get SARS-CoV-2, and can become prone to develop a severe range of symptoms associated with COVID-19.^12^-^14^ This finding has generated a huge debate among researchers to consider the importance of elaborated work in this area.^15^ Recent advances in this direction have shown that individuals with blood type O exhibit a lower chance of encountering severe symptoms as compared to those with blood group Type-A.^16^

Numerous studies have linked specific ABO phenotypes to a higher risk of developing COVID-19. ABO has four fundamental phenotypes: O, A, B, and AB. While weak variants of the blood Group-B phenotype are uncommon, there are numerous different blood Group-A subgroups in which RBCs tend to weakly express the A antigen. The immune system develops antibodies against any ABO blood group antigens that are absent from the person’s RBCs. As a result, a person in Group-A will have anti-B antibodies and a person in Group-B will have anti-A antibodies. People with blood type O in their serum will include both anti-A and anti-B antibodies.^17^ Blood Group-AB is the least common, and these individuals will have neither anti-A nor anti-B in their serum.^18^ A Multi-centric retrospective analysis showed critically ill patients with COVID-19 in ICUs predominantly had blood type A Up till now research suggests the most commonly involved blood type remains A for reasons unknown.^19^

**Table-II:** Association of laboratory parameters of Covid-19 with bloods groups

	Total number	Blood Group					P value

	Of Patients	A+ve	AB+ve	B+ve	O-ve	O+ve	
***TLC_Q***							
Raised	103	55(45.8%)	14(11.7%)	28(23.3%)	2(1.7%)	4(3.3%)	0.461
Normal	15	7(5.8%)	1(0.8%)	4(3.3%)	1(0.8%)	2(1.7%)	
Decreased	2	0(0%)	1(0.8%)	1(0.8%)	0(0%)	0(0%)	
***D_Dimer_Q***							
Normal	32	17(14.2%)	3(2.5%)	9(7.5%)	1(0.8%)	2(1.7%)	0.946
Raised	88	45(37.5%)	13(10.8%)	24(20%)	2(1.7%)	4(3.3%)	
***Ferritin_Q***							
Normal	34	15(12.5%)	3(2.5%)	11(9.2%)	2(1.7%)	3(2.5%)	0.266
Raised	86	47(39.2%)	13(10.8%)	22(18.3%)	1(0.8%)	3(2.5%)	
***LDH_Q***							
Normal	13	4(3.3%)	0(0%)	6(5%)	0(0%)	3(2.5%)	0.005
Raised	107	58(48.3%)	16(13.3%)	27(22.5%)	3(2.5%)	3(2.5%)	
***CRP_Q***							
Normal	45	21(17.5%)	6(5%)	12(10%)	1(0.8%)	4(3.3%)	0.635
Raised	76	41(34.2%)	10(8.3%)	21(17.5%)	2(1.7%)	2(1.7%)	
***Procalcitonin_Q***							
Normal	3	2(1.7%)	1(0.8%)	0(0%)	0(0%)	0(0%)	0.711
Raised	106	60(50%)	15(12.5%)	27.50%	3(2.5%)	6(5%)	

**Table-III:** Association between severity of COVID-19 with clinicopathologic parameters

Parameters	Total No. of Patients	Severity					P value

		Asymptomatic	Mild	Moderate	Severe	Critical	
***Age***							
>50	89	8(6.7%)	8(6.7%)	27(22.5%)	27(22.5%)	19(15.8%)	0.226
≤50	31	7(5.8%)	3(2.5%)	5(4.2%)	11(9.2%)	5(4.2%)	
***Gender***							
Male	64	7(5.8%)	3(2.5%)	20(16.7%)	17(14.2%)	17(14.2%)	0.077
Female	56	8(6.7%)	8(6.7%)	12(10%)	21(17.5%)	7(5.8%)	
***Ethnicity***							
Sindhi Speai	30	5(4.2%)	4(3.3%)	8(6.7%)	8(6.7%)	5(4.2%)	0.687
Urdu	67	6(5%)	6(5%)	20(16.7%)	21(17.5%)	14(11.7%)	
Punjabi	15	2(1.7%)	0(0%)	2(1.7%)	6(5%)	5(4.2%)	
Pathan	8	2(1.7%)	1(0.8%)	2(1.7%)	3(2.5%)	0(0%)	
***Blood Group***							
A+ve	62	4(3.3%)	5(4.2%)	15(12.5%)	24(20%)	14(11.7%)	0.264
AB+ve	16	2(1.7%)	2(1.7%)	2(1.7%)	5(4.2%)	5(4.2%)	
B+ve	33	7(5.8%)	2(1.7%)	13(10.8%)	6(5%)	5(4.2%)	
O-ve	3	0(0%)	1(0.8%)	1(0.8%)	1(0.8%)	0(0%)	
O+ve	6	2(1.7%)	1(0.8%)	1(0.8%)	2(1.7%)	0(0%)	
***DM***							
Yes	61	7(5.8%)	6(5%)	14(11.7%)	20(16.7%)	14(11.7%)	0.845
No	59	8(6.7%)	5(4.2%)	18(15%)	18(15%)	10(8.3%)	
***HTN***							
Yes	71	9(7.5%)	8(6.7%)	17(14.2%)	25(20.8%)	12(10%)	0.583
No	49	6(5%)	3(2.5%)	15(12.5%)	13(10.8%)	12(10%)	
***Fever***							
Yes	66	0(0%)	4(3.3%)	24(20%)	30(25%)	8(15%)	0.001
No	44	15(12.5%)	7(5.8%)	8(6.7%)	8(6.7%)	6(5%)	
***Cough***							
Yes	49	0(0%)	4(3.3%)	12(10%)	24(20%)	9(7.5%)	0.001
No	71	15(12.5%)	7(5.8%)	20(16.7%)	14(11.7%)	15(12.5%)	
***SOB***							
Yes	61	0(0%)	3(2.5%)	8(15%)	31(25.8%)	19(15.8%)	0.001
No	49	15(12.5%)	8(6.7%)	14(11.7%)	7(5.8%)	5(4.2%)	
***Body Ache***							
Yes	17	0(0%)	4(3.3%)	5(4.2%)	6(5%)	2(1.7%)	0.101
No	103	15(12.5%)	7(5.8%)	27(22.5%)	32(26.7%)	22(18.3%)	
***Loss of Taste***							
Yes	4	0(0%)	2(1.7%)	2(1.7%)	0(0%)	0(0%)	0.026
No	116	15(12.5%)	9(7.5%)	30(25%)	38(31.7%)	24(20%)	
***Loss of Smell***							
Yes	6	0(0%)	2(1.7%)	2(1.7%)	2(1.7%)	0(0%)	0.186
No	114	15(12.5%)	9(7.5%)	30(25%)	36(30%)	24(20%)	
***Gen. weakness***							
Yes	28	1(0.8%)	6(5%)	6(5%)	10(8.3%)	5(4.2%)	0.062
No	102	14(11.7%)	5(4.2%)	26(21.7%)	28(23.3%)	19(15.8%)	
***Outcome***							
Discharged	98	15(12.5%)	10(8.3%)	29(24.2%)	31(25.8%)	13(10.8%)	0.001
Death	22	0(0%)	1(0.8%)	3(2.5%)	7(5.8%)	11(9.2%)	

**Table-IV:** Association of severity of COVID-19 with laboratory parameters.

Parameters	Total No: of Patients	Severity					P value

		Asymptomatic	Mild	Moderate	Severe	Critical	
***TLC_Q***							
Raised	103	12(10%)	9(7.5%)	29(24.2%)	32(26.7%)	21(17.5%)	0.402
Normal	15	2(1.7%)	1(0.8%)	3(2.5%)	6(5%)	3(2.5%)	
Decreased	2	1(0.8%)	1(0.8%)	0(0%)	0(0%)	0(0%)	
***D_Dimer_Q***							
Normal	32	10(8.3%)	3(2.5%)	9(7.5%)	7(5.8%)	3(2.5%)	0.003
Raised	88	5(4.2%)	8(6.7%)	23(19.2%)	31(25.8%)	21(17.5%)	
***Ferritin_Q***							
Normal	34	8(6.7%)	4(3.3%)	6(5%)	13(10.8%)	3(2.5%)	0.04
Raised	86	7(5.8%)	7(5.8%)	26(21.7%)	25(20.8%)	21(17.5%)	
***LDH_Q***							
Normal	13	8(6.7%)	1(0.8%)	3(2.5%)	1(0.8%)	0(0%)	0.001
Raised	107	7(5.8%)	10(8.3%)	29(24.2%)	37(30.8%)	24(20%)	
***CRP_Q***							
Normal	44	11(9.2%)	8(6.7%)	12(10%)	9(7.5%)	4(3.3%)	0.001
Raised	76	4(3.3%)	3(2.5%)	20(16.7%)	29(24.2%)	20(16.7%)	
***Procalcitonin_Q***							
Normal	3	0(0%)	1(0.8%)	0(0%)	1(0.8%)	1(0.8%)	0.487
Raised	117	15(12.5%)	10(8.3%)	32(26.7%)	37(30.8%)	23(19.2%)	

Moreover, genetic links with both susceptibility to infection and severity have been reported for the ABO locus.^20^ There are several potential reasons why people with different blood types may experience different outcomes when infected with COVID-19.^21^ Some possibilities include ABO antibodies potentially providing protection against the virus, ABO(H) antigens potentially helping the virus enter host cells, and a higher risk of blood clotting events in individuals with non-O blood types.^22^ These differences in underlying mechanisms may affect various stages of the disease and have implications for the transmission of the virus and the outcome of COVID-19.

Our results align with the findings of Bari A et al.^23^ and Bhattacharjee et al.,^24^ who also identified a significant association between blood Group-A and severe COVID-19. In his detailed analysis, Bhattacharjee further supported this with data indicating a higher frequency of Blood Group-A in patients with severe COVID-19. 24

Our study diverges from the results obtained by Rehman FU et al.,^25^ Hafiz W et al.^26^ and Bryce E Pasko et al.^27^ who found no significant association between blood groups and disease severity. In studies by Barnkob et al.^28^ and Dite et al.^29^ Blood Group-O, individuals were less likely to contract the virus, whereas blood Group-A was associated with an increased risk. However, Shah et al.^30^ found a link between ABO blood group types along with Rh factor antigen (B+ and O+) with the severity of Covid-19 positive patients. Noor et al interestingly found that there is a strong association with susceptibility of contracting COVID-19 in individuals with Blood Group-A not with the severity of infection.^31^ Variations in findings might arise from differences in sample size, population genetics, and geographical region.

Our results are consistent with these findings. While our findings suggest a potential utility of ABO blood group typing as a predictive marker for disease severity, it is crucial to note that other factors such as age, sex, and comorbid conditions can significantly influence disease progression. Further research is necessary to understand the mechanisms underlying this association and its potential implications for disease management and personalized patient care. Future studies might also consider exploring the potential role of ABO blood group in the prognosis of COVID-19 and the implications of blood group typing for vaccine and therapeutic development.

Apart from phenotypic ABO blood groups, genetic variations within the ABO system could influence disease susceptibility and severity, necessitating further research. The study’s findings may inform tailored healthcare approaches, routine screening, risk assessment, vaccine strategies, and therapeutic interventions, presenting significant implications for patient management in Karachi and global COVID-19 understanding.

### Limitations

The limitations of the study are short duration of time and small sample size.

## CONCLUSION

We found a significant association between blood Group-A and severity of Covid-19.Our findings suggest the potential utility of ABO blood group typing as a predictive marker for disease severity, which can contribute to personalized patient management strategies. Further research is necessary to understand the mechanisms underlying this association.
